# Between-Community Low-Income Status and Inclusion in Mandatory Bundled Payments in Medicare’s Comprehensive Care for Joint Replacement Model

**DOI:** 10.1001/jamanetworkopen.2021.1016

**Published:** 2021-03-08

**Authors:** Joshua M. Liao, Qian Huang, Said A. Ibrahim, John Connolly, Deborah S. Cousins, Jingsan Zhu, Amol S. Navathe

**Affiliations:** 1 Department of Medicine, University of Washington, Seattle; 2 Department of Medical Ethics and Health Policy, University of Pennsylvania, Philadelphia; 3 Population Health Sciences, Weill Cornell Medicine, New York, New York; 4 Perelman School of Medicine, University of Pennsylvania, Philadelphia; 5 Corporal Michael J. Cresencz VA Medical Center, Philadelphia, Pennsylvania

## Abstract

This cohort study examines whether communities in Medicare’s Comprehensive Care for Joint Replacement (CJR) Model are representative of others nationwide with respect to residents’ socioeconomic status.

## Introduction

Using a market-level mandate, Medicare’s Comprehensive Care for Joint Replacement (CJR) Model has required urban US hospitals to accept bundled payments for hip and knee surgery episodes. Among metropolitan statistical area (MSA) markets with above-average episode spending (196 of 384 MSAs), Medicare randomly selected 67 for inclusion.^1^ Given the 3% to 4% episode savings and stable quality achieved through CJR, Medicare has reinforced its commitment to MSA market-level mandates, using the approach in the forthcoming Radiation Oncology Model with another mandatory program planned in 2023.^2,3^

One key advantage of mandatory over voluntary programs is mitigating physician or hospital self-selection that could lead to the exclusion of patients with low socioeconomic status (SES).^4^ This advantage can also enhance generalizability of program results, but only if regions in the program do not differ greatly from those not included. However, it remains unclear whether communities in CJR are representative of others nationwide with respect to residents’ SES.

## Methods

This cohort study was approved by the University of Pennsylvania institutional review board with a waiver of informed consent because of the infeasibility of obtaining consent from a large retrospective claims data set. Our analysis followed the Strengthening the Reporting of Observational Studies in Epidemiology (STROBE) reporting guideline.

We measured low SES using Medicaid and Medicare dual eligibility, which federal policy makers consider the most reliable measure of social risk among Medicare beneficiaries.^5^ We used 2016 Medicare data to identify CJR MSA markets and 2010 to 2012 Medicare data to define hospital service areas (HSAs), which are collections of zip codes whose residents receive the majority of hospitalizations from hospitals in that area, as communities within MSAs. We then measured community-level dual-eligibility share, which is the proportion of dual-eligible individuals in HSAs.^6^ The HSA-level dual-eligibility share was clustered at the MSA level, an approach that reflects the fact that although CJR participation was determined at the MSA level, the program incentivized care changes more narrowly among hospitals.

We compared dual-eligibility share and other characteristics between CJR and non-CJR markets using Wilcoxon rank sum tests. We evaluated the association between dual-eligibility share, categorized into quartiles to allow for nonlinear associations, and CJR market status using multivariable linear regression on HSA-level data, controlling for market characteristics (shown in the Table) and clustered at the MSA level.

**Table.** Dual-Eligibility Share and Other Characteristics by CJR and Non-CJR Markets

Characteristic^a^	Non-CJR markets (n = 306 MSAs with 915 HSAs)		CJR markets (n = 67 MSAs with 389 HSAs)		*P* value
	Population, mean (SD), %	Population, median (IQR), %	Population, mean (SD), %	Population, median (IQR), %	
General population					
Sex					
Female	50.7 (1.6)	NA	50.9 (1.3)	NA	.008^b^
Male	49.3 (1.6)	NA	49.1 (1.3)	NA	.008^b^
Race/ethnicity^c^					
White	83.5 (16.3)	NA	79.9 (19.7)	NA	.007^b^
Black	8.0 (11.4)	3.2 (0.9-10.0)	7.9 (10.7)	3.6 (1.5-9.8)	.03^b^
Hispanic	5.4 (10.9)	1.6 (0.7-4.9)	7.2 (11.8)	2.5 (0.8-7.9)	<.001^b^
Asian or Pacific Islander	1.6 (3.5)	0.6 (0.3-1.3)	3.3 (6.8)	0.8 (0.4-2.2)	<.001^b^
Other^d^	1.6 (3.7)	1.1 (0.8-1.6)	1.7 (3.5)	1.2 (0.8-2.0)	.006^b^
Age group, y					
0-64	85.9 (4.2)	NA	85.6 (4.8)	NA	.92
65-74	7.7 (2.2)	NA	7.6 (2.2)	NA	.16
75-84	4.5 (1.5)	NA	4.7 (1.9)	NA	.40
≥85	1.9 (0.7)	NA	2.0 (1.0)	NA	.03^b^
Medicare population					
Beneficiaries, No.	27 616 (36 000)	17 585 (10 174-29 563)	31 133 (39 659)	18 661 (10 416-34 780)	.12
Clinical complexity, No.^e^	8.4 (1.0)	NA	8.3 (0.9)	NA	.06
Medicare Advantage penetration	22.9 (13.3)	NA	30.4 (15.2)	NA	<.001^b^
Osteoarthritis prevalence	2.8 (0.9)	NA	2.7 (0.9)	NA	.006^b^
Hip and knee joint replacement prevalence	1.1 (0.2)	NA	1.0 (0.3)	NA	<.001^b^
Hospital and postacute care facility supply, No.					
Total hospital beds	570 (1117)	267 (129-582)	753 (1355)	303 (150-699)	.01^b^
Inpatient rehabilitation facility beds	35 (84)	10 (0-36)	43 (92)	12 (0-40)	.11
Skilled nursing facility beds	76 (216)	16 (0-75)	80 (188)	20 (0-74)	.18
Total home health agency staff	323 (909)	98 (34-247)	417 (1055)	106 (34-345)	.28
Dual-eligibility share^f^	17.2 (7.2)	NA	17.5 (8.4)	NA	.68

Abbreviations: CJR, Comprehensive Care for Joint Replacement Model; HSA, hospital service area communities; IQR, interquartile range; MSA, metropolitan statistical area market; NA, not applicable.

^a^ Variables shown represent market characteristics used in multivariable regression analyses.

^b^ Denotes significance at *P* < .05.

^c^ Race and ethnicity were based on Medicare claims data definitions, rather than by investigators or participants. These data were included in this analysis as part of a set of market-level variables that could potentially be associated with our exposure and/or outcome. Given historical disparities facing Black and Hispanic individuals, we included an indicator of membership in these 2 groups in regression analyses.

^d^ Includes American Indian/Alaska native, other (designated in claims data), and unknown (designated in claims data).

^e^ Determined by the Elixhauser Mortality Index.

^f^ The dual-eligibility share is 2.8% to 12.3% for quartile 1, 12.3% to 15.9% for quartile 2, 15.9% to 20.6% for quartile 3, and 20.6% to 57.7% for quartile 4.

Analyses were performed using SAS statistical software version 9.4 (SAS Institute). Statistical tests were 2-tailed and significant at α = .05. Data analysis was performed from October 2020 to January 2021.

## Results

Our sample consisted of 67 CJR markets containing 389 HSAs and 306 non-CJR markets containing 915 HSAs (Table). The mean (SD) dual-eligibility share was 17.5% (8.4%) of the population among CJR markets and 17.2% (7.2%) of the population among non-CJR markets. There were small differences between CJR and non-CJR markets with respect to population sex, age, and racial/ethnic mix, as well as other characteristics, such as total number of hospital beds and Medicare Advantage penetration.

In multivariable analysis, market-level dual-eligibility share was inversely associated with the likelihood of being a CJR market (Figure). The probability of being a CJR market decreased from the lowest quartile (quartile 1) of dual-eligibility share to quartile 2 (−4.5 percentage point probability; 95% CI, −8.3 to −0.7 percentage point probability; *P* = .02) and quartile 3 (−8.3 percentage point probability; 95% CI, −14.6 to −2.0 percentage point probability; *P* = .01). An increase from the lowest to highest quartile (quartile 4) of dual-eligibility share was associated with a −14.1 percentage point probability of being a CJR market (95% CI, −22.2 to −6.0 percentage point probability; *P* < .001).

**Figure.**
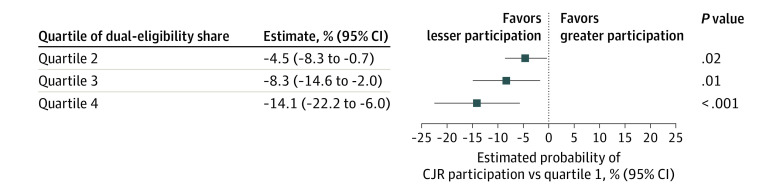
Association Between Dual-Eligibility Share and Comprehensive Care for Joint Replacement (CJR) Program Participation Graph shows that the likelihood of being a CJR market decreases as market dual-eligibility share increases (ie, as quartile increases). The dual-eligibility share is 2.8% to 12.3% for quartile 1, 12.3% to 15.9% for quartile 2, 15.9% to 20.6% for quartile 3, and 20.6% to 57.7% for quartile 4.

## Discussion

Markets that were more likely to have a higher burden of adverse outcomes through social risk factors were less likely to be selected for CJR. A limitation of this study is the observational design; however, this study underscores the need to ensure that expanded or additional market-level mandates do not inadvertently perpetuate SES disparities. Policy makers should urgently address this concern by directly considering community social factors when selecting markets in forthcoming mandatory bundled payment programs.
